# Combination of percutaneous radiofrequency ablation and systemic chemotherapy are effective treatment modalities for metachronous liver metastases from gastric cancer

**DOI:** 10.1007/s10585-013-9606-5

**Published:** 2013-08-22

**Authors:** Jun-Eul Hwang, Seung-Hun Kim, Joon Jin, Ji-Yun Hong, Min-Jee Kim, Sung-Hoon Jung, Hyun-Jeong Shim, Woo-kyun Bae, Eu-Chang Hwang, Jin-Woong Kim, Sang-Soo Shin, Oh Jeong, Young Kyu Park, Sang-Hee Cho, Ik-Joo Chung

**Affiliations:** 1 Department of Hematology and Oncology, Chonnam National University Hwasun Hospital and Medical School, 322 Seoyang-ro, Hwasun-eup, Hwasun-gun, Jeonnam, Kwangju, 519-763 Korea; 2 Department of Urology, Chonnam National University Hwasun Hospital and Medical School, Kwangju, Korea; 3 Department of Diagnostic Radiology, Chonnam National University Hwasun Hospital and Medical School, Kwangju, Korea; 4 Department of Surgery, Chonnam National University Hwasun Hospital and Medical School, Kwangju, Korea

**Keywords:** Radiofrequency ablation, Stomach neoplasms, Metastasis, Chemotherapy

## Abstract

This study evaluated the efficacy of percutaneous radiofrequency ablation (RFA) for the treatment of metachronous liver metastases of gastric cancer. We enrolled a total of 44 patients who underwent percutaneous RFA for the treatment of metachronous liver metastases after resection of a primary gastric adenocarcinoma from January 2002 to November 2011. The primary endpoint of this study was overall survival (OS) and recurrence-free survival (RFS) after RFA. Systemic chemotherapy was combined with RFA in 40 patients; the OS and RFS of the patients with liver-only metastasis who underwent RFA and chemotherapy were 20.9 months (95 % CI 18.4–23.4) and 9.8 months (95 % CI 9.2–10.5), respectively. On multivariate analysis, the factors independently, negatively associated with OS were extrahepatic metastatic lesions (HR 12.6, 95 % CI 3.7–42.9; *p* = 0.001), no chemotherapy (HR 43.3, 95 % CI 7.4–251.3; *p* = 0.001), and tumor number ≥2 (HR 2.6, 95 % CI 1.2–5.9; *p* = 0.015). The factors independently, negatively associated with RFS were extrahepatic metastatic lesions (HR 3.6, 95 % CI 1.6–7.8; *p* = 0.003) and bilobar intrahepatic distribution (HR 3.9, 95 % CI 1.5–9.9; *p* = 0.001). The efficacy of percutaneous RFA for metachronous liver metastases of gastric cancer is limited to patients with a single, unilobar metastasis without extrahepatic metastatic lesions. Combined systemic chemotherapy is very important for the prolongation of OS.

## Introduction

Liver metastasis from gastric cancer (LMGC) has a very poor prognosis and there are no effective treatment modalities [1]. Liver metastases can be found in 5–9 % of patients with gastric cancer, and cancer recurrence and metastatic patterns are associated with locoregional, peritoneal, and hematogenous metastases; all metastatic patterns are found simultaneously in most cases [2–4]. The vast majority of patients with LMGC may in fact have systemic disease. Only a very small subset of patients with LMGC are candidates for surgical resection as the disease is often associated with extrahepatic metastatic lesions: peritoneal dissemination, lymph node metastases, and direct cancer invasion of other organs [1, 4, 5].

The benefits of surgical resection for LMGC are unsatisfactory; systemic chemotherapy is a standard treatment approach for most patients with this disease [1, 6, 7]. Radiofrequency ablation (RFA) is a commonly-used alternative to surgery for tumor ablation [8]. The treatment efficacy of RFA for LMGC, however, remains poorly defined. In this study, we evaluated the efficacy of percutaneous RFA for treatment of metachronous liver metastases of gastric cancer after curative resection of the primary lesion.

## Patients and methods

### Patients

At Chonnam National University Hwasun Hospital in Jeonnam, Korea, a total of 1,342 patients with recurrent or metastatic gastric cancer received palliative chemotherapy between January 2002 and November 2011. The criteria for inclusion in our study of RFA for liver metastases were as follows: (1) histologically confirmed gastric adenocarcinoma; (2) presence of metachronous liver metastases, arising more than 3 months after surgery; (3) percutaneous complete ablation of the liver metastases was feasible; (4) no evidence of anastomotic site recurrence; (5) absence of extrahepatic metastatic lesions (preferable), or presence of minimal extrahepatic metastatic lesions with liver metastases considered suitable for RFA. Of the original 1,342 patients, a total of 229 (17 %) had liver metastases. Of these 229 patients, 185 were not considered eligible for RFA due to tumor growth in multiple liver segments, extensive extrahepatic metastatic lesions, and/or unfavorable clinical conditions such as poor performance status, old age, and cardiopulmonary dysfunction. We prospectively enrolled a total of 44 patients to undergo RFA for the treatment of metachronous liver metastasis after curative margin-negative (R0) resection of the primary gastric adenocarcinoma. All data were prospectively recorded and only the survival data was updated from the cancer registry at the time of analyses. Eastern Cooperative Oncology Group performance status (ECOG PS) was evaluated according to the established criteria. This study was approved by the Institutional Review Board of Chonnam National University Medical School Research Institution (2012-110). The recommendations of the Declaration of Helsinki for biomedical research involving human subjects were followed throughout.

Liver metastases were diagnosed during a pre-programmed follow-up schedule of physical examination, simple chest radiography, abdomal computed tomography (CT), endoscopy, and, if necessary, liver magnetic resonance imaging (MRI). Chest CT and bone scan or positron emission tomography were performed to evaluate for extrahepatic metastatic lesions if liver metastases were detected. A total of five patients received a needle aspiration biopsy just before RFA, as their abdominal CT and MRI scans did not show the typical enhancement pattern of liver metastases. All biopsy results demonstrated metastatic adenocarcinoma.

### RFA technique

Percutaneous hepatic lesion RFA was performed, under local and intravenous anesthesia, by two experienced interventional radiologists with 14 and 6 years of experience, respectively. RFA was performed using an internally-cooled monopolar electrode with a 3 cm active tip (Valleylab, Boulder, CO, USA) and a 150 W radiofrequency generator (Cool-tip RF System, Radionics, Burlington, MA, USA). A standard grounding pad (Valleylab, Boulder, CO, USA) was placed on the patient’s back. All RFA was performed under real-time sonographic guidance with a 1–5 MHz curved probe (LOGIQ E9, GE Healthcare, Milwaukee, WI, USA). Radiofrequency current was applied to each lesion for 12 min at 150 W to create a radius of ablation at least 10 mm larger than the largest tumor diameter. If the tumors were larger than 2.5 cm in greatest diameter, we performed multiple overlapping ablations to cover the entire tumor, with a 5 mm ablative margin. After RFA, the intrahepatic electrode track was cauterized to minimize bleeding and prevent tumoral seeding.

One hour after the initial treatment, contrast-enhanced dynamic CT was performed. When the thickness of the ablative margin was at least 0.5 cm for the index tumor, the treatment was considered finished. When a residual enhanced lesion was seen on dynamic CT, an additional RFA was immediately performed.

### Follow-up

Local therapeutic and technical efficacy was evaluated by contrast-enhanced dynamic CT scanning 1 month after RFA. Clinical tumor recurrence and response was assessed according to the Response Evaluation Criteria in Solid Tumors (RECIST version 1.0) [9], by subsequent CT scanning after every two or three courses of chemotherapy. When patients did not receive chemotherapy, CT scanning was performed every 3 months after RFA, or in the case of clinical suspicion for recurrence.

### Chemotherapy

Peri-procedural systemic chemotherapy was administered to patients just before (*n* = 23) or after (*n* = 17) RFA (within 3 weeks before or after RFA). In patients with no evidence of recurrence, chemotherapy was maintained for 6 months. When follow-up CT showed intrahepatic or extrahepatic recurrence, second-line chemotherapy was initiated or best supportive care was performed, according to the clinical situation. Chemotherapy regimens included a variety of agents such as taxanes, oxaliplatin, irinotecan, cisplatin, 5-fluorouracil (5-FU), and TS-1. Doublet chemotherapy regimens were most commonly used.

### Statistics

Kaplan–Meier analysis was applied to assess clinical factors affecting overall survival (OS) and recurrence-free survival (RFS), and the significance of differences in survival curves was determined by the log-rank test. OS was defined as the period from the date of RFA to the date of death from any cause. RFS was defined as the period from the date of RFA to the date of disease progression or death, whichever occurred first. If neither event had occurred at the time of the last record, the patient was censored at that time. The factors included in univariate survival analysis were age, gender, ECOG PS, tumor size, tumor number, presence of liver-only metastases, presence of extrahepatic metastatic lesions, and intrahepatic tumor distribution (unilobar or bilobar). Multivariate regression analysis using the Cox proportional hazards regression model (stepwise forward procedure), was performed to achieve an adjusted hazard ratio (HR) to determine the prognostic factors for OS and RFS. A 2-tailed *p* < 0.05 was considered significant for all analysis. The SPSS software package, version 19.0 (SPSS Inc., Chicago, IL, USA) was used for statistical analysis.

## Results

### Patient characteristics

The baseline characteristics of the 44 patients are listed in Table 1. All patients had gastric adenocarcinoma and there were no distant metastases identified at the time of initial operation. All patients received curative resection for their gastric cancer, combined with D2 lymph node dissection. The median follow-up time (from surgery to death or last follow-up date) was 31.7 months, with a range of 10.5–57.1 months. The median patient age was 64 years, with a range of 38–83 years. A total of 26 patients (59.1 %) were male, and 18 patients (40.9 %) were female. The median time to development of liver metastases after surgery was 12.9 months (95 % CI 3.9–37.7 months). A total of 13 patients (29.5 %) had simultaneous extrahepatic metastatic lesions: 11 patients (25 %) had metastases to intraabdominal lymph nodes; 1 patient had peritoneal seeding nodules; one patient had a metastatic pleural nodule. A total of 30 patients (68.2 %) had a unilobar intrahepatic distribution, and 14 patients (31.8 %) had bilobar intrahepatic metastatic tumors. A total of 23 patients had a single liver metastasis, 20 patients had 2 liver metastatic tumors, and only 1 patient had 3 liver metastases. The median size of the largest metastatic liver tumor was 2 cm, with a range of 1–2.7 cm. A total of 40 patients (90.9 %) received systemic chemotherapy, and 18 patients (41 %) received second-line chemotherapy. Only four patients did not receive systemic chemotherapy due to patient’s refusal; these patients had liver-only metastases. All patients with extrahepatic metastatic lesions received chemotherapy. The most commonly used chemotherapy regimen was a oxaliplatin and 5-FU doublet (*n* = 16, 40 %). The specific chemotherapy regimens are shown in Table 2.

**Table 1:** Patient characteristics (*n* = 44)

Characteristics	Number of patients (*n* = 44)
Age (years)	
Median (range)	64 (38–83)
Gender	
Male/Female	26/18
ECOG PS	
0/1	20/24
Primary tumor pathologic stage	
II	21
III	23
Histologic grade	
Well differentiated	37
Poorly differentiated	7
Chemotherapy	
Yes/No	40/4
Tumor size (cm)	
Median (range)	2 (1–2.7)
Tumor number	
1	23
≥2	21
Extrahepatic metastatic lesion	
No extrahepatic disease	31
Intraabdominal lymph node	11
Peritoneum	1
Pleura	1
Intrahepatic distribution	
Unilobar	30
Bilobar	14

*ECOG PS* Eastern Cooperative Oncology Group performance status

**Table 2:** Chemotherapy regimens (*n* = 40)

Regimen	Number of patients	%
Oxaliplatin/5-FU	16	40
Irinotecan/5-FU	8	20
Taxane/Cisplatin	12	30
Weekly paclitaxel	2	5
TS-1	2	5
Total	40	100

### Results of RFA

Radiofrequency ablation was technically successful for all tumors. A total of 42 patients showed complete disappearance of tumor enhancement in the contrast-enhanced CT acquired 1 h after treatment. Two patients with small residual tumors underwent immediate additional RFA. There were no significant adverse events. Almost all patients demonstrated mild to moderate abdominal pain and liver enzyme elevations. Both the abdominal pain and liver enzyme elevations resolved with supportive care. A total of three patients suffered from transient Gram-negative bacteremia. These patients were treated with antibiotics: two patients were treated with a third generation cephalosporin, one with a carbapenem. All three recovered without sequelae.

### Survival and prognostic factors

**Fig. 1:**
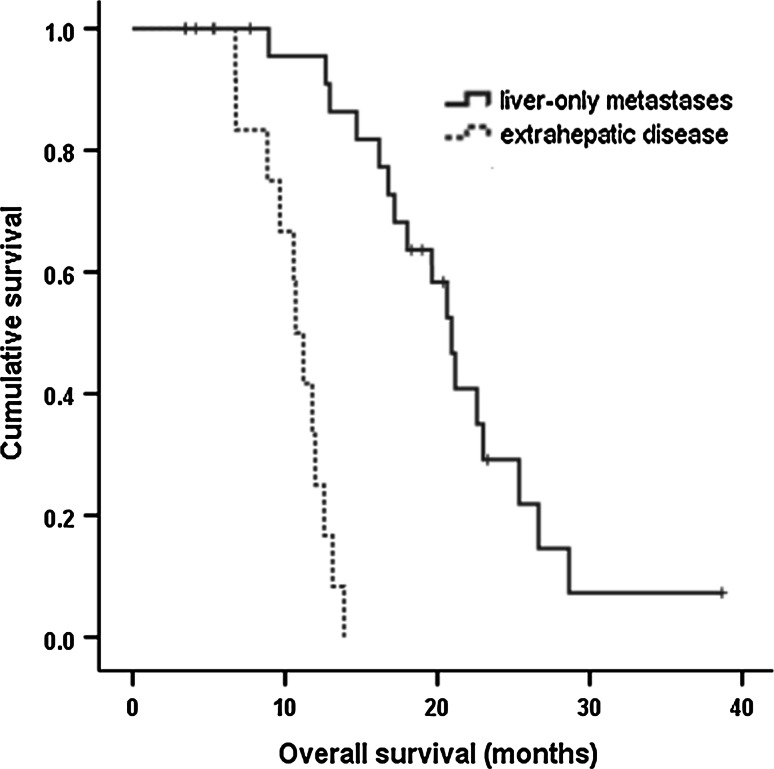
Overall survival curve for patients with liver-only metastases who underwent radiofrequency ablation and chemotherapy (*blue line*) and patients with extrahepatic metastatic lesion who underwent the same treatment (*green line*). (Color figure online)

**Fig. 2:**
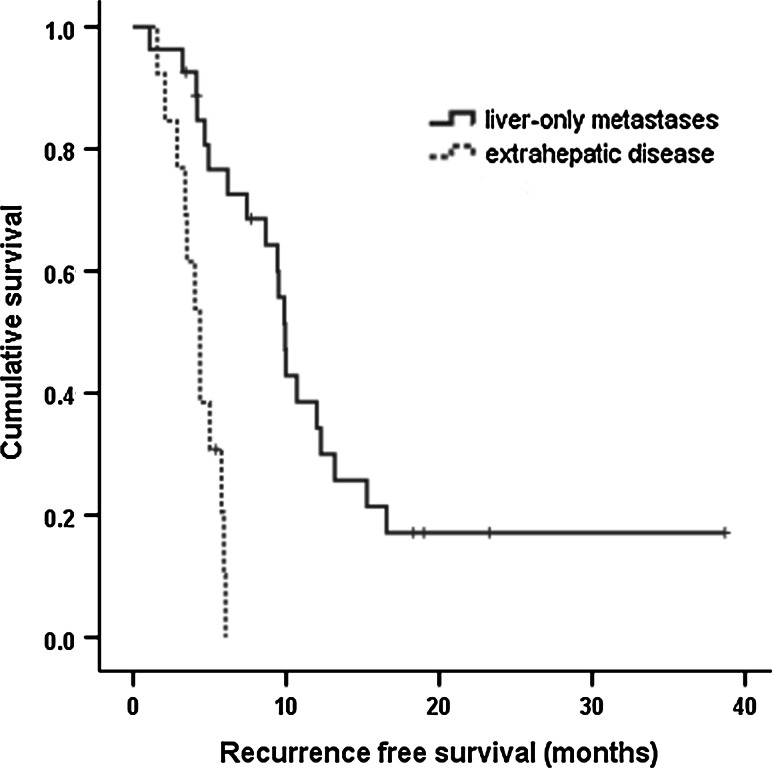
Recurrence-free survival curve for patients with liver-only metastases who underwent radiofrequency ablation and chemotherapy (*blue line*) and patients with extrahepatic metastatic lesion who underwent the same treatment (*green line*). (Color figure online)

The median OS after RFA in all patients was 14.7 months (95 % CI 10.1–19.2). The median RFS after RFA in all patients was 6.1 months (95 % CI 4.2–8.1). The median OS and RFS of patients with liver-only metastases who underwent RFA and chemotherapy were 20.9 months (95 % CI 18.4–23.4) and 9.8 months (95 % CI 9.2–10.5), respectively. The median OS and RFS of patients with extrahepatic metastatic lesions who underwent RFA and chemotherapy were only 11.2 months (95 % CI 9.8–12.5) and 4.3 months (95 % CI 3.8–4.9), respectively. Patients with liver-only metastases who underwent RFA and chemotherapy had a better median OS and RFS than patients with extrahepatic metastatic lesions who underwent the same treatment. The difference was statistically significant (Table 3) (Figs. 1, 2).

**Table 3:** Patients with liver-only metastases who underwent radiofrequency ablation and chemotherapy had a better median overall survival and recurrence-free survival than patients with extrahepatic metastatic lesion who underwent the same treatment (statistically significant)

	Liver-only metastasis with RFA and chemotherapy (*n* = 27)	Liver and extrahepatic disease with RFA and chemotherapy (*n* = 13)	*p* value
mOS (months)	20.9	11.2	0.001
	(95 % CI 18.4–23.4)	(95 % CI 9.8–12.5)	
mRFS (months)	9.8	4.3	0.001
	(95 % CI 9.2–10.5)	(95 % CI 3.8–4.9)	

*mOS* median overall survival, *mRFS* median recurrence-free survival, *RFA* radiofrequency ablation

In our study, in spite of the small number of patients (*n* = 4), patients who did not receive chemotherapy had significantly shorter OS compared with patients receiving chemotherapy (*n* = 40) [8.1 months (95 % CI 7.6–8.6) vs. 16.7 months (95 % CI 12.0–21.4)]. A total of 18 patients received second-line chemotherapy, however second-line chemotherapy did not affect OS [second-line chemotherapy (+) vs. (−), 18.0 vs. 14.7 months, *p* = 0.203].

In univariate analysis, the OS after RFA was significantly shorter for patients with the following clinical factors: no systemic chemotherapy, the presence of extrahepatic metastatic lesions, ≥2 liver metastases, and bilobar intrahepatic distribution. Univariate analysis also demonstrated that the presence of extrahepatic metastatic lesions, ≥2 liver metastases, and bilobar intrahepatic distribution were significantly associated with a shorter RFS after RFA (Table 4).

**Table 4:** Univariate analysis of clinical factors influencing median overall survival and median recurrence-free survival (*n* = 44)

Clinical factors	mOS		mRFS	
	Months (95 % CI)	*p* value	Months (95 % CI)	*p* value
Age (years)				
<64	13.1 (7.1–19.1)	0.473	5.9 (4.4–7.3)	0.642
≥64	18.0 (9.0–26.9)		7.4 (3.0–11.8)	
Gender				
Male	13.1 (11.1–15.0)	0.827	7.3 (4.8–9.7)	0.269
Female	16.1 (12.1–20.2)		6.0 (0.7–11.3)	
ECOG PS				
0	13.8 (11.3–16.3)	0.761	7.3 (4.2–10.3)	0.504
1	17.1 (7.6–26.6)		5.9 (4.5–7.2)	
Primary tumor pathologic stage				
II	16.1 (9.9–22.4)	0.602	7.3 (4.2–10.4)	0.464
III	14.7 (8.8–20.5)		6.0 (2.5–9.4)	
Histologic grade				
Well differentiated	13.8 (9.0–18.7)	0.811	5.9 (3.2–8.5)	0.925
Poorly differentiated	20.6 (11.3–29.8)		9.4 (5.7–13.0)	
Chemotherapy				
Yes	16.7 (12.0–21.4)	0.001	7.4 (3.5–11.3)	0.379
No	8.1 (7.6–8.6)		5.4 (2.5–8.3)	
Tumor size				
<2 cm	16.1 (5.8–26.4)	0.187	7.4 (2.8–1.9)	0.297
≥2 cm	13.1 (7.6–18.6)		5.9 (2.3–9.5)	
Tumor number				
1	20.9 (11.4–30.3)	0.006	9.9 (8.1–11.7)	0.005
≥2	11.8 (8.9–14.6)		4.6 (3.5–5.7)	
Extrahepatic metastatic lesion				
No (liver-only metastasis)	19.6 (14.5–24.7)	0.001	9.5 (7.9–11.0)	0.001
Yes	11.2 (9.8–12.5)		4.3 (3.8–4.9)	
Intrahepatic distribution				
Unilobar	20.9 (13.9–27.9)	0.001	9.8 (9.1–10.6)	0.001
Bilobar	11.2 (8.6–13.7)		4.1 (3.5–4.7)	

*mOS* median overall survival, *mRFS* median recurrence-free survival, *ECOG PS* Eastern Cooperative Oncology Group performance status

Multivariate regression analysis identified the independent negative prognostic factors for OS and RFS (Table 5). The independent negative prognostic factors for OS were the presence of extrahepatic metastatic lesions (HR 12.6, 95 % CI 3.7–42.9; *p* = 0.001), ≥2 liver metastases (HR 2.6, 95 % CI 1.2–5.9; *p* = 0.015), and no systemic chemotherapy (HR 43.3, 95 % CI 7.4–251.3; *p* = 0.001). The independent negative prognostic factors for RFS were the presence of extrahepatic metastatic lesions (HR 3.6, 95 % CI 1.6–7.8; *p* = 0.003), and intrahepatic bilobar distribution (HR 3.9, 95 % CI 1.5–9.9; *p* = 0.001).

**Table 5:** Multivariate analysis of factors influencing overall survival and recurrence-free survival

Factors	Hazard ratio (95 % CI)	*p* value
OS		
Extrahepatic metastatic lesion	12.6 (3.7–42.9)	0.001
Number of liver metastasis (≥2)	2.6 (1.2–5.9)	0.015
No chemotherapy	43.3 (7.4–251.3)	0.001
RFS		
Extrahepatic metastatic lesion	3.6 (1.6–7.8)	0.003
Intrahetpatic distribution (bilobar)	3.9 (1.5–9.9)	0.001

*OS* overall survival, *RFS* recurrence-free survival

## Discussion

Recurrent or metastatic gastric cancer, including the presence of liver metastases has a very poor prognosis, and most oncologists recommend systemic chemotherapy. According to recent reports, the median OS of patients treated with combination chemotherapy, with or without a targeted agent such as trastuzumab, is about 13 months [6, 10, 11].

Liver resection for metastatic tumors is performed mainly in patients with primary colorectal cancer, with resection rates of 20–30 % and a 5-year survival rate of 25–85 % [12, 13]. In contrast, liver resection for LMGC is a rarely-performed procedure. The rate of hepatic resection in LMGC ranges from only 0.5 to 2.3 % [2, 14]. Most patients with LMGC remain incurable due to bilobar, multinodular tumor spread, gross peritoneal dissemination, diffuse metastases to distant lymph nodes, or unresectable local recurrences [4, 15–17]. In a recently published review, 19 studies were analyzed to compare survival periods following hepatic resection for LMGC. The median survival for all 436 patients was 17 months, and the 5-year survival was 26.5 % [18]. Several authors have reported better OS in patients with metachronous liver metastasis than in those with synchronous disease after hepatic resection [19–21]. Ambiru et al. [21] reported significantly longer survival in patients with metachronous metastasis (5-year survival 29 %) than in those with synchronous disease (5-year survival 6 %). However, Sakamoto et al. [2] reported no significant difference in survival between synchronous and metachronous metastasis after hepatic resection (19.2 vs. 21.4 months, *p* = 0.84). In our study, we found a median OS of 20.9 months for those patients who underwent RFA and chemotherapy for liver-only metastases; this is comparable to the results for surgical resection.

After detecting a liver recurrence, oncologists have to be extremely cautious about patient selection for surgical resection, so as to avoid an inadequate or extensive operation in a high-risk patient whose disease is incurable by resection. Sakamoto et al. [1] reported that unilobar metastases and/or a tumor <4 cm in diameter may be indications for surgical resection. The number of liver metastases can be another significant prognostic factor. Gannon et al. [22] reported that, from a tumor biology and behavior perspective, RFA would not be effective for more than five metastases. Shirabe et al. [23] also reported that the presence of more than three liver metastases was an independent poor prognostic factor, based on observations in patients after liver resection of LMGC. In our study, the independent negative prognostic factors for OS were presence of extrahepatic metastatic lesions, number of liver metastases (≥2), and no systemic chemotherapy.

Recurrent liver tumors are common after curative hepatic resection for gastric metastases, occurring in about two-thirds of patients [7, 24]. This high recurrence rate might suggest the presence of occult intrahepatic metastases, even at the time of the hepatectomy [1]. In our study, a total of 27 patients (61.4 %) developed intrahepatic recurrence after RFA.

The efficacy of adjuvant chemotherapy after liver resection of LMGC has not been fully evaluated [25, 26]. Chemotherapy after liver resection of LMGC may be beneficial in terms of the high risk of recurrence, and the same may apply to RFA. In our study, in spite of the small number of patients (*n* = 4) who did not receive chemotherapy, these patients had significantly shorter OS compared with those who did undergo chemotherapy.

In Korea, it has been common practice for patients who fail first-line palliative chemotherapy to receive second-line chemotherapy and Kang et al. [27] reported that salvage chemotherapy (second- or third-line chemotherapy) in advanced gastric cancer resulted in significant prolongation of survival when compared with best supportive care. In our study, second-line chemotherapy did not affect OS. We propose that this was due to the small number of patients of this study.

Radiofrequency ablation has been used for the treatment of primary and secondary hepatic malignancies, particularly in hepatocellular carcinoma and colorectal cancer, and its use is steadily increasing. Compared with hepatic resection, RFA is relatively less invasive, resulting in fewer complications, a lower mortality rate, a shorter hospital stay, and a lower treatment cost [8, 28]. The role of RFA in LMGC is not clearly defined. Several reports have demonstrated the beneficial local control effect of RFA and the potential additional benefit of chemotherapy, however, these reports featured a very small and heterogeneous patient population [14, 25, 29].

Considering the high recurrence rate of this disease and the early clinical recovery after RFA compared with surgery, the combined treatment of systemic chemotherapy and RFA may be a potential therapeutic strategy for metachronous liver metastases after resection of the primary gastric cancer. More early administration of chemotherapy may be possible after RFA rather than after surgery.

## Conclusion

Combination of percutaneous RFA and systemic chemotherapy may be effective treatment modalities for patients with a single, unilobar metachronous LMGC without extrahepatic metastatic disease.
